# α-methylacyl-CoA racemase (AMACR) expression in chordomas differentiates them from chondrosarcomas

**DOI:** 10.1038/srep21277

**Published:** 2016-02-18

**Authors:** Sarit Aviel-Ronen, Oranit Zadok, Aya Vituri, Dvora Nass, Ignat Schwartz, Camila Avivi, Iris Barshack

**Affiliations:** 1 Department of Pathology, Sheba Medical Center, 52621 Tel Hashomer, Israel; 2 Talpiot Medical Leadership Program, Sheba Medical Center, Israel; 3 Department of Statistics and Operations Research, Tel Aviv University, Tel Aviv, Israel; 4 Sackler Faculty of Medicine, Tel Aviv University, Tel Aviv, Israel

## Abstract

**Aims**: Chordomas and chondrosarcomas are malignant mesenchymal tumours with overlapping morphological and immunohistochemical (IHC) characteristics. Our aim was to evaluate the IHC expression of α-methylacyl-CoA racemase (AMACR/P504S), β-catenin and E-cadherin in chordomas relative to chondrosarcomas and assess the utility of these markers for differential diagnosis. **Methods**: Archival sections of 18 chordomas, 19 chondrosarcomas and 10 mature cartilage samples were immunostained and scored for AMACR, β-catenin and E-cadherin and the relative differential capacity of each marker was calculated. In addition, AMACR mRNA level was assessed in 5 chordomas by RT-PCR and evaluated by comparative C_T_ method. **Results**: AMACR and β-catenin stained 88.9% and 94.1% of the chordomas respectively, 21.1% and 10.5% of the chondrosarcomas correspondingly and none of the mature cartilage samples. E-cadherin stained positively 82.4% of the chordomas, 36.8% of the chondrosarcomas and 42.9% of the mature cartilage cases. Both AMACR and β-catenin showed statistically significant difference between chordomas and chondrosarcomas (p < 0.001 for both), unlike E-cadherin. AMACR was detected at the mRNA level. **Conclusions**: AMACR is expressed in most of the chordomas but only in a minority of chondrosarcomas. AMACR may serve as IHC marker of chordoma with differentiating ability comparable to that of β-catenin.

Chordomas are locally aggressive malignant tumours of notochordal origin that typically arise in bones of the base of skull, the vertebral bodies and the sacrococcygeal bone. They account for 1–4% of all primary malignant bone tumours^1^ and for 40% of all primary sacral tumours^2^. They are almost twice as common in men as in women (1.8:1) and their incidence peaks after the fifth decade of life^3^. Chordoma cells are characterized by epithelioid pale vacuolated cytoplasm (physaliphorous cells) with mild to moderate nuclear atypia, but severe atypia can appear when sarcomatoid features supervene. They tend to grow in sheets, cords or float within abundant myxoid stroma, creating lobules separated by fibrous bands. Chordoma cells typically show positive immunostaining for S-100 protein and epithelial cell markers like cytokeratins (CK) (specifically CK 8, 18 and 19) and epithelial membrane antigen (EMA)^3^,^4^,^5^. Brachyury, a transcription factor, known to be involved in notochordal development, is highly specific to chordomas among soft tissue tumours, with the exception of hemangioblastoma^3^,^5^,^6^. Cytogenetically, chordomas are characterized by deletion of the chromosome 9p21 locus containing the CDKN2A/2B genes (70% of cases), and gain of the chromosome 6q27 locus containing the T gene (brachyury) present in about half of the chordomas. Copy number gains of 7q33 and 7p12 loci are also common events^3^,^6^,^7^. Radical surgical resection is the treatment of choice and when incomplete resection is suspected, adjuvant radiotherapy is required^5^.

The pathological differential diagnosis of chordomas includes chondrosarcoma, metastatic carcinoma, myxopapillary ependymoma and chordoid meningioma^4^. Immunohistochemical (IHC) stains are useful adjunct diagnostic tool: metastatic carcinomas are CKs positive but stain negatively for S-100, myxopapillary ependymomas are positive for S-100 but co-express glial fibrillary acidic protein (GFAP) while chordoid meningiomas are positive for EMA but negative for CK and GFAP^4^. The introduction of brachyuruy stain has resolved the problem of distinguishing chordoma from chondrosarcoma as well as other tumours with similar morphology. Yet, to date this antibody is still accessible to a limited number of laboratories. Thus differentiating chordomas from chondrosarcomas remains challenging for many practitioners, particularly when handling small biopsies as CK expression may be focal in the chondroid component^5^.

Chondrosarcoma is the third most common malignant tumour of the bone (20% of the cases) after myeloma and osteosarcoma. The bones of the pelvis are the most common skeletal site of chondrosarcoma but rarely, chondrosarcoma may involve the spine and the base of the skull. Likewise chordomas, chondrosarcomas are slightly more common in men than in women and peak at the same age range^3^.

Histologically, chondrosarcomas invade the bone, creating irregularly shaped lobules with some fibrous bands. The chondrocytes are surrounded by abundant cartilaginous/myxoid matrix. The morphological features vary with tumour grade and more aggressive tumours have higher degrees of cellularity and nuclear pleomorphism^2^,^3^. Cytogenetically chondrosarcomas show recurrent losses of 9p21 (containing the CDKN2A gene) and 13q and gains of 8q24 and 12q13^3^,^8^. Recently mutations in IDH1/IDH2 (in about half of the cases) and COL2A1 (37% of cases) have been found^3^,^9^,^10^. Unlike chordomas, low-grade chondrosarcomas have 85% 10 year survival and are treated only but conservative surgery^5^.

Therefore, the distinction between chrodomas and chondrosarcomas has important clinical implications. Since the two entities share some clinical and morphological features, the differentiation between them at times may be difficult. In fact, gene expression analysis has found that of all cartilaginous neoplasms chordomas are most closely related to chondrosarcomas^5^,^11^, as both produce abundant extracellular matrix rich in type II collagen, cartilage oligomeric matrix protein, SOX9 and many other proteins^5^,^12^. Chordomas and chondrosarcomas also may react similarly with IHC stains: occasional chordomas are EMA and/or CK-negative while about 8% of chondrosarcoma stain with EMA and CK as well as S-100^4^. Various studies attempted to identify proteins whose expression differentiates these tumours, among them are the adhesion molecules β-catenin and E-cadherin. Both these proteins are reported to stain more frequently chordomas than chondrosarcomas^13^,^14^,^15^ but with inconsistency of results^4^.

E-cadherin and β-catenin belong to the Wnt signaling pathway and react with each other^16^. Recently a link between β-catenin gene mutation and overexpression of the enzyme α-methylacyl-CoA racemase (AMACR, also known as P504S) was found in hepatocellular carcinomas, suggesting that the latter is regulated by β-catenin-mediated signaling^17^. AMACR, is an enzyme that plays a crucial role in the beta-oxidation of branched-chain fatty acids and fatty acid derivatives^18^. It was initially identified as an immuno-marker of prostatic cancer, but its overexpression has been subsequently shown in a variety of other tumours including liver, kidney and colorectal cancers^17^,^18^,^19^,^20^.

In view of the reported association of chordomas with β-catenin staining and β-catenin with AMACR expression, we hypothesized that chordomas are associated with overexpression of AMACR. To test our hypothesis we have assessed by IHC stains the expression of E-cadherin, β-catenin and AMACR in a cohort of chordomas and compared it to a set of chondrosarcomas. Next we evaluated mRNA level of AMACR. To conclude, we compared the capacity of these three IHC markers to differentiate chordomas from chondrosarcomas.

## Methods

### The study cohort

The study was approved by the institutional Helsinki committee. We have identified in the archive of the Department of Pathology at the Sheba Medical Center 18 chordomas, 19 chondrosarcomas and 10 cases of mature cartilage tissue. All samples were resected from 1994–2014. The diagnosis of all cases was revised by the study pathologist (SAR). Guided by routine hematoxylin & eosin (HE) stained slides, representative formalin-fixed paraffin embedded (FFPE) block from each case was chosen for IHC study.

### IHC staining

FFPE blocks were sectioned at 4 μm and a positive control was added to each slide. All IHC staining was fully calibrated on a Benchmark XT staining module (Ventana Medical Systems Inc., USA). Briefly, after sections were dewaxed and rehydrated, a CC1 Standard Benchmark XT pretreatment for antigen retrieval (Ventana Medical Systems) was selected for all immunostains: AMACR (P504S, ready to use, Biocare Medical, USA), β-catenin, (1:50, Cell Marque, USA), E-cadherin, (1:25, Life Technologies, Invitrogen, USA) and brachyury (1:200, Santa Cruz Biotechnology Inc., USA). Detection of AMACR, β-catenin and E-cadherin was performed with iView DAB Detection Kit (Ventana Medical Systems Inc., USA) and of brachyury with ultraView DAB Detection Kit (Ventana Medical Systems Inc., USA). Slides were counterstained with hematoxylin (Ventana Medical Systems Inc., USA) on an automated stainer and dehydrated in ethanol solutions (70%, 96%, and 100%) for one minute each. Before cover-slipping, sections were cleared in xylene for 2 minutes and mounted with Entellan (Surgipath, Germany).

### Microscopic evaluation and photography

All slides were examined under Olympus BX50 microscope. IHC staining was scored by the study pathologist (SAR). The cut-off for positivity was ≥10% of cells with positive staining. AMACR showed granular cytoplasmic staining, E-Cadherin had membranous and cytoplasmic staining and β-catenin was scored for cytoplasmic staining. The intensity of staining was scored as well but was not included due to its lack of statistical significance. Microscopic images were taken using Olympus DP71 camera and Cell^B software.

### AMACR mRNA expression by RT-PCR

Total RNA was extracted from FFPE tumour samples of 8 chordomas and 3 chondrosarcomas with relatively large tumour tissue required for the procedure. Total RNA extracted from FFPE tumour sample of prostatic adenocarcinoma was used as a reference (calibrator) sample. The RNA was extracted using the RNeasy FFPE kit (Qiagen, Valencia, USA) according to the manufacturer’s instructions. Reverse transcription of total RNA was performed using the qScript cDNA Synthesis kit (Quanta Biosciences, Gaithersburg, USA) and includes the use of a mixture of random and Oligo (dT) primers and recombinant Moloney-murine leukemia virus RT as described in the manufacturer’s protocol. Quantitative RT-PCR amplification was performed with Platinum® SYBR® Green qPCR SuperMix (Life Technologies, Invitrogen, USA) in a 16 μl reaction volume containing 2 μl of cDNA products as a template. The expression of three housekeeping genes GAPDH, β-actin and HPRT was used for normalization. Primers were designed using the primer express software (GAPDH) and the primer 3 software (β-actin and HPRT). All of the primer pairs designed were verified for specificity by comparing to the human RefSeq database of mRNAs (by using Blast search for short nearly exact matches). The primer sequences used were as follow: GAPDH (F: 5′-ACCCACTCCTCCACCTTTGA-3′; R: 5′-CTGTTGCTGTAGCCAAATTCGT-3′), β-actin (F: 5′-GTCCACCTTCCAGCAGATGT-3′; R: 5′-AAAGCCATGCCAATCTCATC-3′), HPRT (F-5′-ACGTCTTGCTCGAGATGTGA-3′; R: 5′-AATCCAGCAGGTCAGCAAAG-3′), AMACR (F: 5′-GGCAAGGGTCAGGTCATTG-3′; R: 5′-AGGTGCTCCACCATCCAAC-3′)^21^. Analysis of the relative gene expression was performed using the 2^−ΔΔCT^ method^22^. All reactions were performed in triplicates. For every sample the average C_T_ for each gene was calculated from the triplicate readings. Next, for each sample the geometric mean of the averaged C_T_ of the housekeeping genes was calculated and served as the endogenous reference. Finally, 2^−ΔΔCT^ method was applied using the readings of the prostatic adenocarcinoma sample for calibration.

### Statistics

Statistical analysis was carried out on R Statistical Software, using the Fisher’s exact test, one-sided for evaluation of each staining, exact conditional test, two-sided for comparison of differentiating capacity of AMACR to β-catenin, unpaired two-sided Kruskal–Wallis test for age assessment and Fisher’s exact test, two-sided for gender study. Statistical significance was defined in general as P < 0.05, and the alpha level was adjusted for multiple comparisons using the Bonferroni correction for evaluation of each staining and for comparison of differentiating capacity of AMACR to β-catenin: alpha = 0.05/13 = 0.0038.

## Results

### The study cohort

The characteristics of the study cohort are summarized in Table 1. Chordoma patients included 5 females and 13 males with a mean age at surgery of 59.2 years (age range 18–82 years, *Std* 13.98). The chordomas were located in the chest wall (1), cranium (6) and along the spine (11). Chondrosarcoma patients consisted of 8 females and 11 males with a mean age at surgery of 45.7 years (age range 25–73, *Std* 15.45). The chondrosarcomas were located in the chest wall (2), femur (4), arm (6), pelvis (4) and spine (3). Among the mature cartilage patients were 8 females and 2 males with a mean age at surgery of 53.7 years (age range 3–86, *Std* 32.17). Mature cartilage samples came from a variety of anatomic sites (5) and from femoral head (5). There was significant statistical dominance of age of the chordoma group over the chondrosarcoma group (*p* = 0.011), but no other significant statistical dominance of age between the study groups (*p* = 0.62 chordoma vs. mature cartilage, *p* = 0. 55 chondrosarcoma vs. mature cartilage). As for gender prevalence, both the chordoma and chondrosarcoma groups contained more males than females with no significant difference between the two groups (*p* = 0.49, *odds ratio* = 1.85). In comparison with mature cartilage there is statistically significant difference only for chordoma (chordoma vs. mature cartilage *p* =* *0.016, *odds ratio* = 9.415; chondrosarcoma vs. mature cartilage *p* = 0.11, *odds ratio* = 5.18).

### IHC staining

Brachyury immunostain confirmed the diagnosis of all cases in our cohort as it stained positively all the chordomas (aside from one case in which tissue was exhausted) and none of the chondrosarcoma or mature cartilage samples.

**Figure 1:**
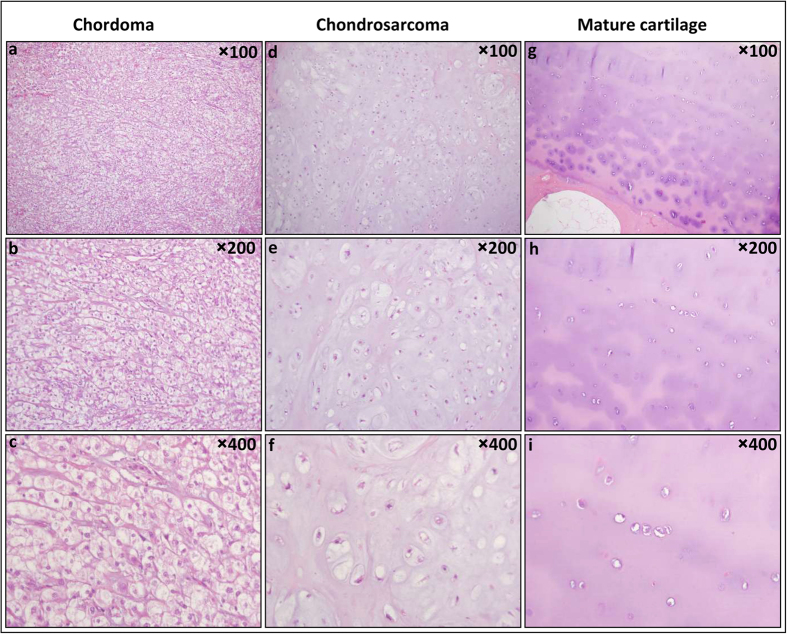
Chordoma, chondrosarcoma and mature cartilage. Chordoma cells have epithelioid pale vacuolated cytoplasm (physaliphorous cells), show mild nuclear atypia and float within abundant myxoid stroma. Chondrosarcoma chondrocytes reside within lacunae, are often binucleated and surrounded by abundant cartilaginous/myxoid matrix. Their cellularity and nuclear atypia increase with grade. Mature cartilage is characterized by abundant matrix containing relatively sparse chondrocytes located in discrete lacunaes, showing no nuclear atypia. Chordoma (**a–c**); Chondrosarcoma (**d–f**) and Mature cartilage (**g–i**) HE X100, X200 and X400.

**Figure 2:**
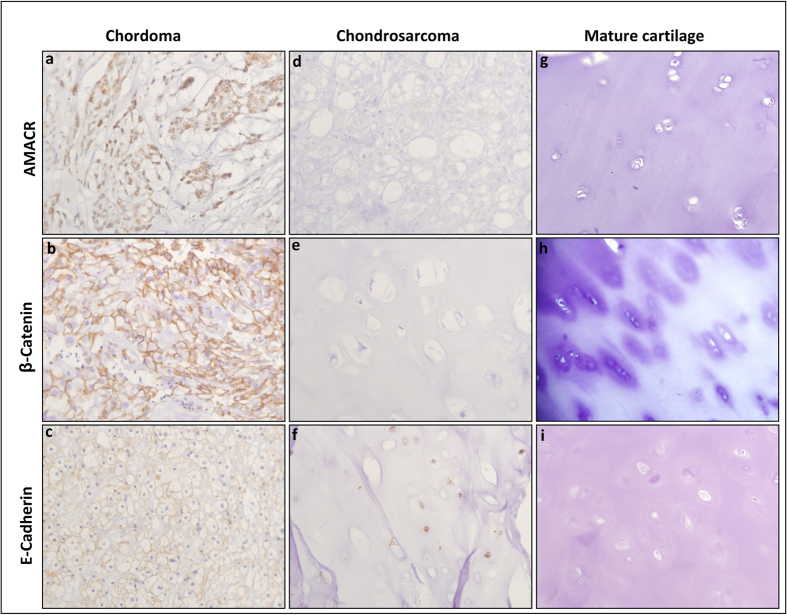
IHC staining of AMACR, β-Catenin and E-cadherin in chordoma, chondrosarcoma and mature cartilage. AMACR and β-catenin show cytoplasmic staining while E-cadherin has membranous and cytoplasmic staining. Chordoma: (**a**) Positive staining of AMACR; (**b**) Positive staining of β-catenin; (**c**) Positive staining of E-cadherin. Chondrosarcoma (**d**) Negative staining of AMACR; (**e**) Negative staining of β-catenin; (**f**) Positive staining of E-cadherin. Mature cartilage: (**g**) Negative staining of AMACR; (**h**) Negative staining of β-catenin; (**i**) Negative staining of E-cadherin. All images at X400.

Thereafter we compared the percentage of cases that were positively stained by AMACR, β-catenin and E-cadherin in the three study groups. Figures 1 and 2 show respectively representative HE and IHC stains images of the different study groups. The results of the IHC study are detailed in Table 2.

AMACR stained positively 88.9% of the chordomas, only 21.1% of the chondrosarcomas and none of the mature cartilage cases. The percentage of positively stained cases was significantly higher in chordoma compared to chondrosarcoma (*p* = 3.839e-05, *odds ratio* = 26.2) and to mature cartilage (*p* = 5.029e-06, *odds ratio* = Inf). However, no significant difference was found for AMACR between the percentage of positively stained cases in chondrosarcoma and mature cartilage (*p* = 0.16).

β-catenin staining showed in our cohort only cytoplasmic staining pattern. No sample has shown nuclear staining. β-catenin stained positively 94.1% of the chordomas, only 10.5% of the chondrosarcomas and none of the mature cartilage cases. Likewise AMACR, the percentage of β-catenin positively stained cases was significantly higher in chordoma compared to chondrosarcoma (*p* = 3.224e-07, *odds ratio* = 100.5) and to mature cartilage (*p* = 2.311e-05, *odds ratio* = Inf). No significant difference was found for β-catenin between the percentage of positively stained cases in chondrosarcoma and mature cartilage (*p* = 0.53).

E-cadherin, stained positively 82.4% of the chordomas, 36.8% of the chondrosarcomas and 42.9% of the mature cartilage cases. Although the percentage of E-cadherin positively stained cases was higher in chordoma compared to chondrosarcoma (*p* = 0.007, *odds ratio* = 7.5), this difference did not reach the minimal level required (0.0038) for statistical significance following the Bonferroni correction for multiple comparisons. No difference in E-cadherin staining was found between chordoma and mature cartilage (*p* = 0.08) or between chondrosarcoma and mature cartilage (*p* = 0.77).

Our results did not show significant difference between the ability of AMACR and β-catenin staining to differentiate chordomas from chondrosarcomas (*p* = 0.74) and chordomas from mature cartilage (*p* = 1).

We were unable to retrieve all planed IHC results. Tissue was exhausted from one of the chordomas and few mature cartilage samples repeatedly fell off the slides due to the tissue nature and the special conditions required for the IHC staining. The missing data is presented as “Not applicable” in Table 2. As there is no connection between the fact a sample is categorized as “Not applicable” and its putative IHC characteristics, these samples were excluded from the analysis presented in Table 2.

### AMACR mRNA expression by RT-PCR

**Figure 3:**
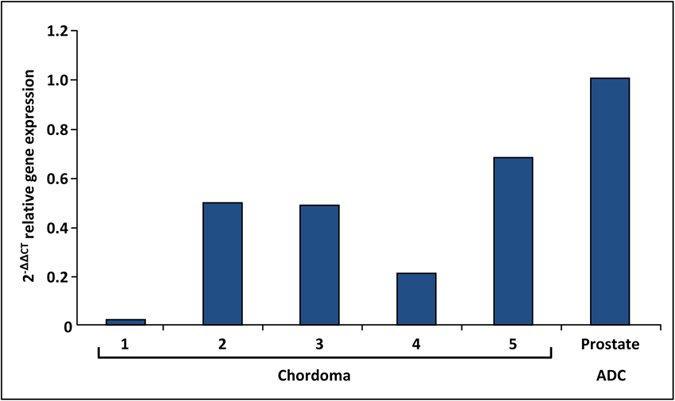
AMACR mRNA relative expression. Relative expression of AMACR mRNA as measured in five chordoma cases and reference sample of prostate adenocarcinoma, using the quantitation-comparative C_T_ analysis.

Following the IHC study that found presence of AMACR protein in the majority of the chordomas, we attempted to explore AMACR mRNA level in the same tumour samples. Unfortunately we have encountered profound technical difficulties in extracting mRNA from FFPE tumour blocks of chordomas and chondrosarcomas and thereafter evaluating their level by RT-PCR technique. Many of the samples in our cohort contained too little tissue to extract RNA. We attempted to extract RNA from 8 chordomas and 3 chondrosarcomas, but were successful in only 5 cases of chordoma. No chondrosarcoma or mature cartilage case had enough tissue and RNA for RT-PCR analysis. In all 5 successful chordoma cases, we have found the presence of AMACR mRNA. All 5 cases showed positive staining for AMACR. The results of the analysis of the relative gene expression, performed using the 2^−ΔΔCT^ method, are presented in Fig. 3.

## Discussion

Our study is the first to report of AMACR expression in chordomas. We have identified AMACR presence in chordomas at both the protein level by IHC stains and at the mRNA level by RT-PCR. We have shown that AMACR is differentially expressed in chordomas and chondrosarcoma. We also found that AMACR ability as a differentiating IHC marker is comparable to that of β-catenin and superior to E-cadherin.

The characteristics of our cohort are similar to the described in the literature for chordomas and chondrosarcomas. The continuous search for IHC markers that would allow a better distinction between chordomas and chondrosarcomas is still relevant due to the existing overlap between the IHC profiles of the available markers. During the last decade few differentiating IHC markers have been suggested. In view of the epithelioid features that chordoma cells display, different adhesion molecules have been studied as IHC markers of chordomas^4^,^13^,^14^,^15^. The most studied adhesion molecules were E-cadherin and β-catenin. Naka *et al*. reported that 86.6% of the chordomas stained positively for β-catenin and 73.3% for E-cadherin^13^, while Mori *et al*. found that all chordomas in their cohort stained positively for E-cadherin^14^. However, subsequent studies have shown lower rates of positive staining. Horiguchi *et al*. found that only 37.5% and 68.8% of the chordomas showed positive staining for β-catenin and E-cadherin respectively^15^ and Cho *et al*. reported that only 7.1% and 14.2% of the chordomas showed positive staining for β-catenin and E-cadherin respectively^4^. With such relatively rare expression of these adhesion molecules Cho *et al*. have concluded that β-catenin and E-cadherin have little value in the differential diagnosis with chondrosarcoma. Instead, they suggested D2-40 as a promising new IHC marker for differential diagnosis of tumours with chordoid morphology as none of the chordomas they have studied showed positive staining for D2-40 while all chondrosarcomas were positively stained. Our study showed results within the described range for β-catenin and E-cadherin. In our cohort 94.1% of the chordomas were positive for β-catenin and 82.4% for E-cadherin. Both markers stained more frequently chordomas than chondrosarcomas. However, only β-catenin reached the minimal level required for statistical significance following the Bonferroni correction for multiple comparisons (*p* = 3.224e-07 for β-catenin, *p* = 0.007 for E-cadherin, alpha = 0.0038).

β-catenin has a role in two pathways involved in tumourigenesis. First, it affects cell adhesion and suppresses tumour invasion through its interaction with E-cadherin at the cell surface that causes recruitment of α-catenin, which in turn binds the intracellular actin cytoskeleton. Second, it is a key mediator in the Wnt signaling pathway which regulates cell proliferation and differentiation. A multi-protein complex that includes adenomatous polyposis coli (APC) and glycogen synthase kinase-3 β (GSK3 β) promotes the degradation of β-catenin thus controls the levels of free β-catenin in cells. Activation of Wnt pathway leads to an inactivation of GSK3 β, which allows β-catenin levels to rise in the cytoplasm, and also to translocate to the nucleus where it activates Wnt-related transcription factors and genes^23^. Mutations in this multi-protein complex or in β-catenin itself that lead to stabilization of the β-catenin protein and to dysregulated activation of the Wnt pathway can be detected by IHC as either cytoplasmic or nuclear staining^3^,^23^. In our cohort β-catenin showed only cytoplasmic staining pattern, suggesting that dysregulated activation of the Wnt pathway may exist.

AMACR is known to be overexpressed in variety of tumours including prostatic adenocarcinoma, hepatocellular carcinoma, renal cell carcinoma and colorectal cancers^17^,^18^,^19^,^20^,^24^. Interestingly, all of these tumours also show overexpression of β-catenin^25^,^26^,^27^,^28^. Recently, Sekine *et al*.^17^ have shown that AMACR overexpression in hepatocellular carcinoma was associated with β-catenin gene (CTNNB1) mutations. They have suggested that AMACR expression may be regulated by β-catenin-mediated signaling. If proven to be true, it is possible that our new finding of AMACR expression in chordomas is also associated with the Wnt pathway. As so far no mutations in β-catenin gene have been identified in chordomas, this hypothesis demands further investigation.

In our current study we have shown for the first time that chordomas express AMACR both at the protein as well as the mRNA level. Moreover, we have found that AMACR may serve as IHC marker of chordoma, assisting in the differential diagnosis with chondrosarcoma. AMACR differentiating utility appeared to be comparable with that of β-catenin.

Our attempts to study AMACR at the mRNA level yielded confirmation of its presence. Yet, in view of the technical problems that we have encountered, leading to narrowing of the cohort, a further study, preferably using fresh frozen tissue is recommended.

AMACR should be used for differential diagnosis of chordomas from chondrosarcomas in combination with the classical stains EMA and CKs. This panel will cover the reported possibility of negative expression in chordomas and positive expression in chondrosarcoma. Likewise CKs, AMACR may be focal and therefore the use of AMACR as part of a panel of stains is advised especially when handling small biopsies.

Brachyury is highly specific marker for chordomas, therefore it is recommended for the work out of differential diagnosis with chondrosarcomas. Accordingly, we used brachyury to confirm the diagnosis of all samples in our cohort. However, since the sole clinical indication for use of brachyury is to identify chordomas, many laboratories choose not to include it in their routine repository due to economical considerations. AMACR, on the other hand, that has varied diagnostic uses is available at most laboratories. Thus its higher accessibility compensates for the lower specificity relative to brachyury.

Last but not least, the differential diagnosis of chordoma includes metastatic carcinoma, aside from chondrosarcoma. In cases suspected for chordoma, where the possibility of metastatic carcinoma of prostatic, hepatic, renal or colorectal origin is considered, AMACR and β-catenin IHC stains cannot assist the differential diagnosis since all of these tumours express both markers. In these cases the differential diagnosis requires the use of other markers such as S-100 and other tumour specific proteins according to the presumed origin (e.g PSA for prostatic carcinoma, glypican 3 for hepatocellular carcinoma, PAX8 for renal carcinoma and CDX-2 for colorectal carcinoma). Moreover, brachyury has been recently found to be expressed in a number of carcinomas including hepatocellular carcinoma, lung, colon and breast carcinomas^29^,^30^,^31^,^32^. Hence, neither brachyury nor AMACR can be used for the differential diagnosis of chordomas from any of these tumours.

In conclusion, our study is the first to show that AMACR is expressed in most of the chordomas but only in a minority of chondrosarcomas. Thus, AMACR may serve as IHC marker of chordoma in combination with the classical stains EMA and CKs, as it shows differentiating ability comparable to that of β-catenin. Nonetheless, more studies are needed to substantiate our results and to identify the specific role of AMACR in chordoma.

## Additional Information

**How to cite this article**: Aviel-Ronen, S. *et al*. α-methylacyl-CoA racemase (AMACR) expression in chordomas differentiates them from chondrosarcomas. *Sci. Rep*. **6**, 21277; doi: 10.1038/srep21277 (2016).

## Figures and Tables

**Table 1:** Characteristics of the study cohort.

Diagnosis	Chordoma	Chondrosarcoma	Mature Cartilage	*p-value*
Number of cases	18	19	10	
Age (years)RangeMean (*Std*)	18–8259.2 (13.98)	25–7345.7 (15.45)	3–8653.7 (32.17)	*0.011**0.62***0.55
GenderMen; WomenRatio	13; 52.6:1	11; 81.4:1	2; 81:2	*0.49**0.016***0.11
Location (number of cases)	Chest wall (1)Cranium (6)Spine (11)	Chest wall (2)Femur (4)Spine (3)Arm (6)Pelvis (4)	Various (5)Femur (5)	

*chordoma compared to chondrosarcoma, **chordoma compared to mature cartilage, ***chondrosarcoma compared to mature cartilage. Statistical analysis was carried out on R Statistical Software using the unpaired two-sided Kruskal–Wallis test for age assessment and Fisher’s exact test, two-sided for gender study. Statistical significance was defined as P < 0.05.

**Table 2:** IHC results in chordoma, chondrosarcoma and mature cartilage.

Diagnosis		Chordoma	Chondrosarcoma	Mature Cartilage	*p-value*
Number of cases (%)		18	19	10	
AMACR staining	Positive	16 (88.9)	4 (21.1)	0 (0)	*<0.001
	Negative	2 (11.1)	15 (78.9)	10 (100)	**<0.001
	NA	0	0	0	***0.16
β-catenin staining	Positive	16 (94.1)	2 (10.5)	0 (0)	*<0.001
	Negative	1 (5.9)	17 (89.5)	7 (100)	**<0.001
	NA	1	0	3	***0.53
E-cadherin staining	Positive	14 (82.4)	7 (36.8)	3 (42.9)	*0.007
	Negative	3 (17.6)	12 (63.2)	4 (57.1)	**0.08
	NA	1	0	3	***0.77

P < 0.05, alpha level (adjusted for multiple comparisons, Bonferroni correction) = 0.0038. *chordoma compared to chondrosarcoma, **chordoma compared to mature cartilage, ***chondrosarcoma compared to mature cartilage. NA–Not applicable (see comment at IHC staining section of the Results). Statistical analysis was carried out on R Statistical Software using the Fisher’s exact test, one-sided for evaluation of each staining. Statistical significance was defined as P < 0.05 and corrected to P < 0.0038 for multiple comparisons.

## References

[b1] McPherson C. M. . Metastatic disease from spinal chordoma: a 10-year experience. J. Neurosurg. Spine 5, 277–280 (2006). 17048762 10.3171/spi.2006.5.4.277

[b2] Ropper A. E. . Primary vertebral tumors: a review of epidemiologic, histological and imaging findings, part II: locally aggressive and malignant tumors. Neurosurgery 70, 211–219; discussion 219 (2012). 21768918 10.1227/NEU.0b013e31822d5f17

[b3] Fletcher C. D. M., Bridge J. M., Hogendoorn P. C. W. & Mertens F. Tumours of Soft Tissue and Bone, (IARC Press, Lyon, 2013).

[b4] Cho H. Y. . Immunohistochemical comparison of chordoma with chondrosarcoma, myxopapillary ependymoma, and chordoid meningioma. Appl. Immunohistochem. Mol. Morphol. 17, 131–138 (2009). 19521276 10.1097/PAI.0b013e3181866a13

[b5] Vujovic S. . Brachyury, a crucial regulator of notochordal development, is a novel biomarker for chordomas. J. Pathol. 209, 157–165 (2006). 16538613 10.1002/path.1969

[b6] Presneau N. . Role of the transcription factor T (brachyury) in the pathogenesis of sporadic chordoma: a genetic and functional-based study. J. Pathol. 223, 327–335 (2011). 21171078 10.1002/path.2816

[b7] Szuhai K., Cleton-Jansen A. M., Hogendoorn P. C. & Bovee J. V. Molecular pathology and its diagnostic use in bone tumors. Cancer Genet. 205, 193–204 (2012). 22682618 10.1016/j.cancergen.2012.04.001

[b8] Schrage Y. M. . Central chondrosarcoma progression is associated with pRb pathway alterations: CDK4 down-regulation and p16 overexpression inhibit cell growth *in vitro*. J. Cell Mol. Med. 13, 2843–2852 (2009). 18624751 10.1111/j.1582-4934.2008.00406.x PMC4498940

[b9] Amary M. F. . IDH1 and IDH2 mutations are frequent events in central chondrosarcoma and central and periosteal chondromas but not in other mesenchymal tumours. J. Pathol. 224, 334–343 (2011). 21598255 10.1002/path.2913

[b10] Tarpey P. S. . Frequent mutation of the major cartilage collagen gene COL2A1 in chondrosarcoma. Nat. Genet. 45, 923–926 (2013). 23770606 10.1038/ng.2668 PMC3743157

[b11] Henderson S. R. . A molecular map of mesenchymal tumors. Genome Biol. 6, R76 (2005). 16168083 10.1186/gb-2005-6-9-r76 PMC1242211

[b12] Schwab J. H. . Chordoma and chondrosarcoma gene profile: implications for immunotherapy. Cancer Immunol. Immunother. 58, 339–349 (2009). 18641983 10.1007/s00262-008-0557-7 PMC3426285

[b13] Naka T. . Immunohistochemical analysis of E-cadherin, alpha-catenin, beta-catenin, gamma-catenin, and neural cell adhesion molecule (NCAM) in chordoma. J. Clin. Pathol. 54, 945–950 (2001). 11729215 10.1136/jcp.54.12.945 PMC1731331

[b14] Mori K., Chano T., Kushima R., Hukuda S. & Okabe H. Expression of E-cadherin in chordomas: diagnostic marker and possible role of tumor cell affinity. Virchows Arch. 440, 123–127 (2002). 11964040 10.1007/s004280100525

[b15] Horiguchi H. . Expression of cell adhesion molecules in chordomas: an immunohistochemical study of 16 cases. Acta Neuropathol. 107, 91–96 (2004). 14608467 10.1007/s00401-003-0770-6

[b16] Tian X. . E-cadherin/beta-catenin complex and the epithelial barrier. J. Biomed. Biotechnol. 2011, 567305 (2011). 22007144 10.1155/2011/567305 PMC3191826

[b17] Sekine S., Ogawa R., Ojima H. & Kanai Y. Overexpression of alpha-methylacyl-CoA racemase is associated with CTNNB1 mutations in hepatocellular carcinomas. Histopathology 58, 712–719 (2011). 21457159 10.1111/j.1365-2559.2011.03798.x

[b18] Jiang Z. . Expression of alpha-methylacyl-CoA racemase (P504s) in various malignant neoplasms and normal tissues: astudy of 761 cases. Hum. Pathol. 34, 792–796 (2003). 14506641 10.1016/s0046-8177(03)00268-5

[b19] Li W. . Significance of overexpression of alpha methylacyl-coenzyme. A racemase in hepatocellular carcinoma. J. Exp. Clin. Cancer Res. 27, 2 (2008). 18577240 10.1186/1756-9966-27-2 PMC2438330

[b20] Went P. T., Sauter G., Oberholzer M. & Bubendorf L. Abundant expression of AMACR in many distinct tumour types. Pathology 38, 426–432 (2006). 17008281 10.1080/00313020600922470

[b21] Zielie P. J. . A novel diagnostic test for prostate cancer emerges from the determination of alpha-methylacyl-coenzyme a racemase in prostatic secretions. J. Urol. 172, 1130–1133 (2004). 15311056 10.1097/01.ju.0000133560.87118.4d

[b22] Livak K. J. & Schmittgen T. D. Analysis of relative gene expression data using real-time quantitative PCR and the 2(-Delta Delta C(T)) Method. Methods 25, 402–408 (2001). 11846609 10.1006/meth.2001.1262

[b23] Ng T. L. . Nuclear beta-catenin in mesenchymal tumors. Mod. Pathol. 18, 68–74 (2005). 15375433 10.1038/modpathol.3800272

[b24] Zhou M., Roma A. & Magi-Galluzzi C. The usefulness of immunohistochemical markers in the differential diagnosis of renal neoplasms. Clin. Lab Med. 25, 247–257 (2005). 15848735 10.1016/j.cll.2005.01.004

[b25] Bismar T. A., Humphrey P. A., Grignon D. J. & Wang H. L. Expression of beta-catenin in prostatic adenocarcinomas: a comparison with colorectal adenocarcinomas. Am. J. Clin. Pathol. 121, 557–563 (2004). 15080308 10.1309/4470-49GV-52H7-D258

[b26] Inagawa S. . Expression and prognostic roles of beta-catenin in hepatocellular carcinoma: correlation with tumor progression and postoperative survival. Clin. Cancer Res. 8, 450–456 (2002). 11839663

[b27] Guo L. . The complementary role of beta-catenin in diagnosing various subtypes of renal cell carcinomas and its up-regulation in conventional renal cell carcinomas with high nuclear grades. Oncol. Rep. 8, 521–526 (2001). 11295073 10.3892/or.8.3.521

[b28] Elzagheid A. . Nuclear beta-catenin expression as a prognostic factor in advanced colorectal carcinoma. World J. Gastroenterol. 14, 3866–3871 (2008). 18609711 10.3748/wjg.14.3866 PMC2721444

[b29] Du R., Wu S., Lv X., Fang H. & Kang J. Overexpression of brachyury contributes to tumor metastasis by inducing epithelial-mesenchymal transition in hepatocellular carcinoma. J. Exp. Clin. Cancer Res. 33, 105 (2014). 25499255 10.1186/s13046-014-0105-6 PMC4279691

[b30] Kilic N. . Brachyury expression predicts poor prognosis at early stages of colorectal cancer. Eur. J. Cancer 47, 1080–1085 (2011). 21220197 10.1016/j.ejca.2010.11.015

[b31] Palena C. . Overexpression of the EMT driver brachyury in breast carcinomas: association with poor prognosis. J. Natl. Cancer Inst. 106 (2014). 10.1093/jnci/dju054 PMC4568990 24815864

[b32] Roselli M. . Brachyury, a driver of the epithelial-mesenchymal transition, is overexpressed in human lung tumors: an opportunity for novel interventions against lung cancer. Clin. Cancer Res. 18, 3868–3879 (2012). 22611028 10.1158/1078-0432.CCR-11-3211 PMC3472640

